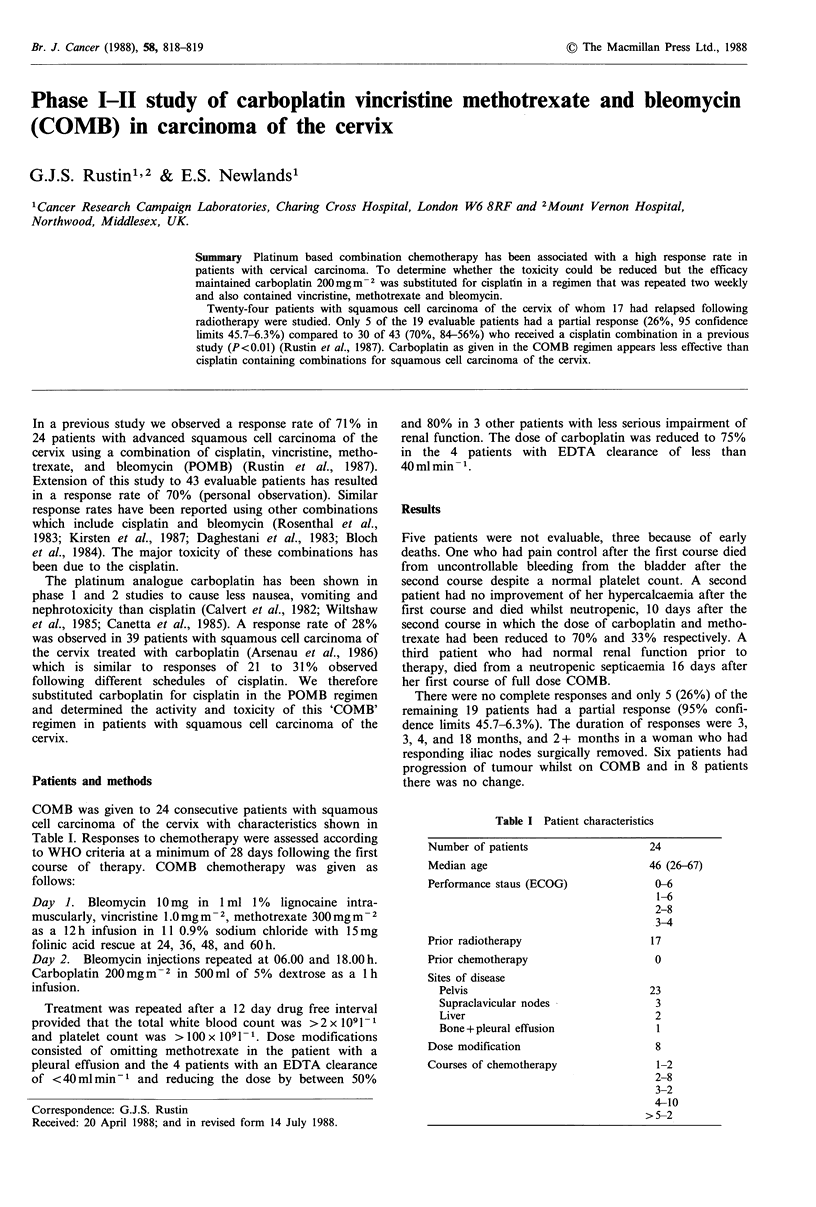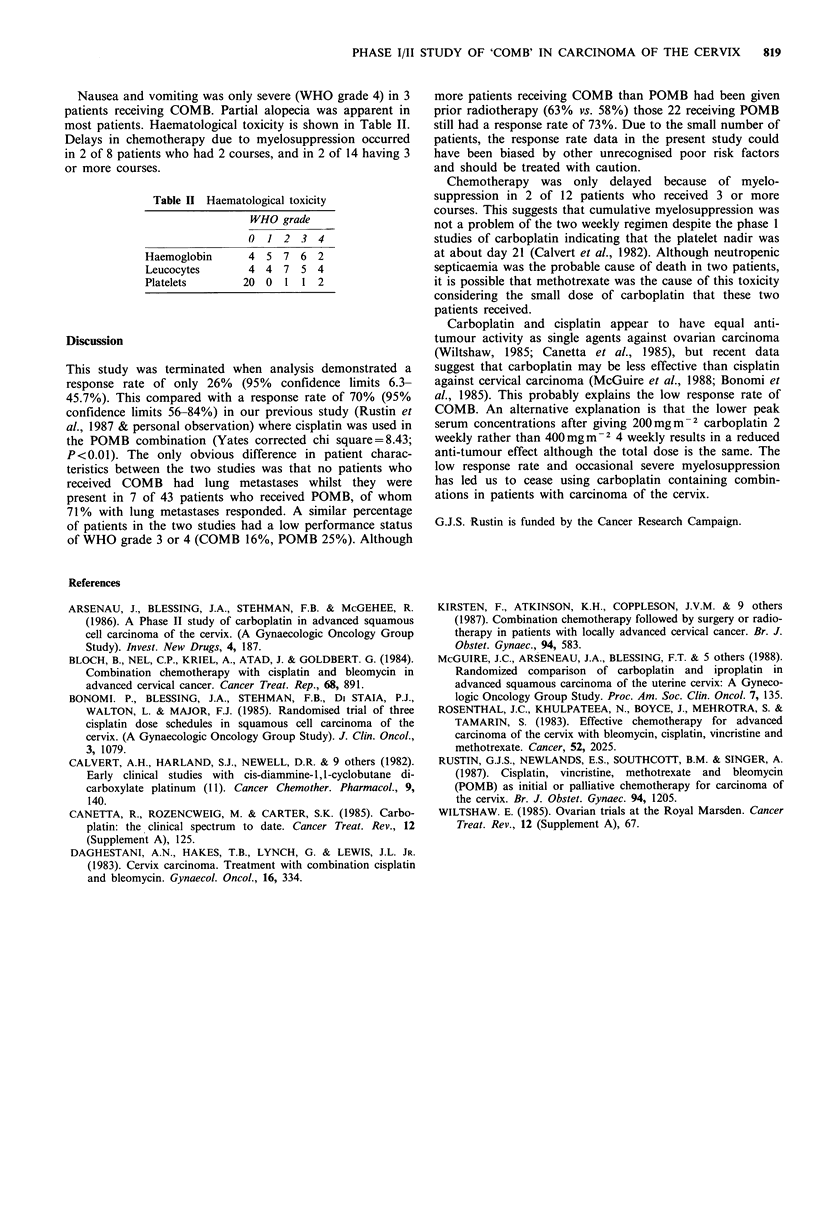# Phase I-II study of carboplatin vincristine methotrexate and bleomycin (COMB) in carcinoma of the cervix.

**DOI:** 10.1038/bjc.1988.317

**Published:** 1988-12

**Authors:** G. J. Rustin, E. S. Newlands

**Affiliations:** Cancer Research Campaign Laboratories, Charing Cross Hospital, London, UK.

## Abstract

Platinum based combination chemotherapy has been associated with a high response rate in patients with cervical carcinoma. To determine whether the toxicity could be reduced but the efficacy maintained carboplatin 200 mg m-2 was substituted for cisplatin in a regimen that was repeated two weekly and also contained vincristine, methotrexate and bleomycin. Twenty-four patients with squamous cell carcinoma of the cervix of whom 17 had relapsed following radiotherapy were studied. Only 5 of the 19 evaluable patients had a partial response (26%, 95 confidence limits 45.7-6.3%) compared to 30 of 43 (70%, 84-56%) who received a cisplatin combination in a previous study (P less than 0.01) (Rustin et al., 1987). Carboplatin as given in the COMB regimen appears less effective than cisplatin containing combinations for squamous cell carcinoma of the cervix.


 © The Macmillan Press Ltd., 1988

### Phase I–II study of carboplatin vincristine methotrexate and bleomycin (COMB) in carcinoma of the cervix

G.J.S. Rustin[1,2] & E.S. Newlands[1]

[1]Cancer Research Campaign Laboratories, Charing Cross Hospital, London W6 8RF and [2]Mount Vernon Hospital, Northwood, Middlesex, UK.

**Summary** Platinum based combination chemotherapy has been associated with a high response rate in patients with cervical carcinoma. To determine whether the toxicity could be reduced but the efficacy maintained carboplatin $200\,mg\,m^{-2}$ was substituted for cisplatin in a regimen that was repeated two weekly and also contained vincristine, methotrexate and bleomycin.

Twenty-four patients with squamous cell carcinoma of the cervix of whom 17 had relapsed following radiotherapy were studied. Only 5 of the 19 evaluable patients had a partial response (26%, 95 confidence limits 45.7–6.3%) compared to 30 of 43 (70%, 84–56%) who received a cisplatin combination in a previous study ($P<0.01$) (Rustin *et al.*, 1987). Carboplatin as given in the COMB regimen appears less effective than cisplatin containing combinations for squamous cell carcinoma of the cervix.

In a previous study we observed a response rate of 71% in 24 patients with advanced squamous cell carcinoma of the cervix using a combination of cisplatin, vincristine, methotrexate, and bleomycin (POMB) (Rustin *et al.*, 1987). Extension of this study to 43 evaluable patients has resulted in a response rate of 70% (personal observation). Similar response rates have been reported using other combinations which include cisplatin and bleomycin (Rosenthal *et al.*, 1983; Kirsten *et al.*, 1987; Daghestani *et al.*, 1983; Bloch *et al.*, 1984). The major toxicity of these combinations has been due to the cisplatin.

The platinum analogue carboplatin has been shown in phase 1 and 2 studies to cause less nausea, vomiting and nephrotoxicity than cisplatin (Calvert *et al.*, 1982; Wiltshaw *et al.*, 1985; Canetta *et al.*, 1985). A response rate of 28% was observed in 39 patients with squamous cell carcinoma of the cervix treated with carboplatin (Arsenau *et al.*, 1986) which is similar to responses of 21 to 31% observed following different schedules of cisplatin. We therefore substituted carboplatin for cisplatin in the POMB regimen and determined the activity and toxicity of this 'COMB' regimen in patients with squamous cell carcinoma of the cervix.

### Patients and methods

COMB was given to 24 consecutive patients with squamous cell carcinoma of the cervix with characteristics shown in Table I. Responses to chemotherapy were assessed according to WHO criteria at a minimum of 28 days following the first course of therapy. COMB chemotherapy was given as follows:

*Day 1.* Bleomycin 10 mg in 1 ml 1% lignocaine intramuscularly, vincristine $1.0\,mg\,m^{-2}$, methotrexate $300\,mg\,m^{-2}$ as a 12 h infusion in 1 l 0.9% sodium chloride with 15 mg folinic acid rescue at 24, 36, 48, and 60 h.
*Day 2.* Bleomycin injections repeated at 06.00 and 18.00 h. Carboplatin $200\,mg\,m^{-2}$ in 500 ml of 5% dextrose as a 1 h infusion.

Treatment was repeated after a 12 day drug free interval provided that the total white blood count was $>2\times10^9\,l^{-1}$ and platelet count was $>100\times10^9\,l^{-1}$. Dose modifications consisted of omitting methotrexate in the patient with a pleural effusion and the 4 patients with an EDTA clearance of $<40\,ml\,min^{-1}$ and reducing the dose by between 50% and 80% in 3 other patients with less serious impairment of renal function. The dose of carboplatin was reduced to 75% in the 4 patients with EDTA clearance of less than $40\,ml\,min^{-1}$.

Correspondence: G.J.S. Rustin
Received: 20 April 1988; and in revised form 14 July 1988.

### Results

Five patients were not evaluable, three because of early deaths. One who had pain control after the first course died from uncontrollable bleeding from the bladder after the second course despite a normal platelet count. A second patient had no improvement of her hypercalcaemia after the first course and died whilst neutropenic, 10 days after the second course in which the dose of carboplatin and methotrexate had been reduced to 70% and 33% respectively. A third patient who had normal renal function prior to therapy, died from a neutropenic septicaemia 16 days after her first course of full dose COMB.

There were no complete responses and only 5 (26%) of the remaining 19 patients had a partial response (95% confidence limits 45.7–6.3%). The duration of responses were 3, 3, 4, and 18 months, and 2+ months in a woman who had responding iliac nodes surgically removed. Six patients had progression of tumour whilst on COMB and in 8 patients there was no change.

**Table I** Patient characteristics

| | |
|---|---|
| Number of patients | 24 |
| Median age | 46 (26–67) |
| Performance staus (ECOG) | 0–6 |
| | 1–6 |
| | 2–8 |
| | 3–4 |
| Prior radiotherapy | 17 |
| Prior chemotherapy | 0 |
| Sites of disease | |
| Pelvis | 23 |
| Supraclavicular nodes | 3 |
| Liver | 2 |
| Bone + pleural effusion | 1 |
| Dose modification | 8 |
| Courses of chemotherapy | 1–2 |
| | 2–8 |
| | 3–2 |
| | 4–10 |
| | >5–2 |

Nausea and vomiting was only severe (WHO grade 4) in 3 patients receiving COMB. Partial alopecia was apparent in most patients. Haematological toxicity is shown in Table II. Delays in chemotherapy due to myelosuppression occurred in 2 of 8 patients who had 2 courses, and in 2 of 14 having 3 or more courses.

**Table II** Haematological toxicity

| | *WHO grade* | | | | |
|---|---|---|---|---|---|
| | *0* | *1* | *2* | *3* | *4* |
| Haemoglobin | 4 | 5 | 7 | 6 | 2 |
| Leucocytes | 4 | 4 | 7 | 5 | 4 |
| Platelets | 20 | 0 | 1 | 1 | 2 |

### Discussion

This study was terminated when analysis demonstrated a response rate of only 26% (95% confidence limits 6.3–45.7%). This compared with a response rate of 70% (95% confidence limits 56–84%) in our previous study (Rustin *et al.*, 1987 & personal observation) where cisplatin was used in the POMB combination (Yates corrected chi square = 8.43; $P<0.01$). The only obvious difference in patient characteristics between the two studies was that no patients who received COMB had lung metastases whilst they were present in 7 of 43 patients who received POMB, of whom 71% with lung metastases responded. A similar percentage of patients in the two studies had a low performance status of WHO grade 3 or 4 (COMB 16%, POMB 25%). Although more patients receiving COMB than POMB had been given prior radiotherapy (63% *vs.* 58%) those 22 receiving POMB still had a response rate of 73%. Due to the small number of patients, the response rate data in the present study could have been biased by other unrecognised poor risk factors and should be treated with caution.

Chemotherapy was only delayed because of myelosuppression in 2 of 12 patients who received 3 or more courses. This suggests that cumulative myelosuppression was not a problem of the two weekly regimen despite the phase 1 studies of carboplatin indicating that the platelet nadir was at about day 21 (Calvert *et al.*, 1982). Although neutropenic septicaemia was the probable cause of death in two patients, it is possible that methotrexate was the cause of this toxicity considering the small dose of carboplatin that these two patients received.

Carboplatin and cisplatin appear to have equal antitumour activity as single agents against ovarian carcinoma (Wiltshaw, 1985; Canetta *et al.*, 1985), but recent data suggest that carboplatin may be less effective than cisplatin against cervical carcinoma (McGuire *et al.*, 1988; Bonomi *et al.*, 1985). This probably explains the low response rate of COMB. An alternative explanation is that the lower peak serum concentrations after giving $200\,mg\,m^{-2}$ carboplatin 2 weekly rather than $400\,mg\,m^{-2}$ 4 weekly results in a reduced anti-tumour effect although the total dose is the same. The low response rate and occasional severe myelosuppression has led us to cease using carboplatin containing combinations in patients with carcinoma of the cervix.

G.J.S. Rustin is funded by the Cancer Research Campaign.

### References

ARSENAU, J., BLESSING, J.A., STEHMAN, F.B. & McGEHEE, R. (1986). A Phase II study of carboplatin in advanced squamous cell carcinoma of the cervix. (A Gynaecologic Oncology Group Study). *Invest. New Drugs*, **4**, 187.

BLOCH, B., NEL, C.P., KRIEL, A., ATAD, J. & GOLDBERT. G. (1984). Combination chemotherapy with cisplatin and bleomycin in advanced cervical cancer. *Cancer Treat. Rep.*, **68**, 891.

BONOMI. P., BLESSING, J.A., STEHMAN, F.B., Di STAIA, P.J., WALTON, L. & MAJOR, F.J. (1985). Randomised trial of three cisplatin dose schedules in squamous cell carcinoma of the cervix. (A Gynaecologic Oncology Group Study). *J. Clin. Oncol.*, **3**, 1079.

CALVERT, A.H., HARLAND, S.J., NEWELL, D.R. & 9 others (1982). Early clinical studies with cis-diammine-1,1-cyclobutane dicarboxylate platinum (11). *Cancer Chemother. Pharmacol.*, **9**, 140.

CANETTA, R., ROZENCWEIG, M. & CARTER, S.K. (1985). Carboplatin: the clinical spectrum to date. *Cancer Treat. Rev.*, **12** (Supplement A), 125.

DAGHESTANI, A.N., HAKES, T.B., LYNCH, G. & LEWIS, J.L. Jr. (1983). Cervix carcinoma. Treatment with combination cisplatin and bleomycin. *Gynaecol. Oncol.*, **16**, 334.

KIRSTEN, F., ATKINSON, K.H., COPPLESON, J.V.M. & 9 others (1987). Combination chemotherapy followed by surgery or radiotherapy in patients with locally advanced cervical cancer. *Br. J. Obstet. Gynaec.*, **94**, 583.

McGUIRE, J.C., ARSENEAU, J.A., BLESSING, F.T. & 5 others (1988). Randomized comparison of carboplatin and iproplatin in advanced squamous carcinoma of the uterine cervix: A Gynecologic Oncology Group Study. *Proc. Am. Soc. Clin. Oncol.* **7**, 135.

ROSENTHAL, J.C., KHULPATEEA, N., BOYCE, J., MEHROTRA, S. & TAMARIN, S. (1983). Effective chemotherapy for advanced carcinoma of the cervix with bleomycin, cisplatin, vincristine and methotrexate. *Cancer*, **52**, 2025.

RUSTIN, G.J.S., NEWLANDS, E.S., SOUTHCOTT, B.M. & SINGER, A. (1987). Cisplatin, vincristine, methotrexate and bleomycin (POMB) as initial or palliative chemotherapy for carcinoma of the cervix. *Br. J. Obstet. Gynaec.* **94**, 1205.

WILTSHAW. E. (1985). Ovarian trials at the Royal Marsden. *Cancer Treat. Rev.*, **12** (Supplement A), 67.